# The Seasonal Natural History of the Ant, *Dolichoderus mariae*, in Northern Florida

**DOI:** 10.1673/031.009.0201

**Published:** 2009-02-06

**Authors:** Kristina O. Laskis, Walter R. Tschinkel

**Affiliations:** Department of Biological Science, Florida State University, Tallahassee, FL 32306-4370

**Keywords:** polygyny, colony structure, foraging, polydomy, territoriality, reproductive cycle, monomorphism, flatwoods, mating flights, exudates feeding, wiregrass, nest architecture

## Abstract

*Dolichoderus mariae* Forel, (Hymenoptera: Formicidae) is an uncommon, monomorphic but locally abundant, reddish-brown ant of peculiar nesting habits, whose range includes most of the eastern USA. In north Florida the ant excavates soil under wiregrass clumps or other plants with fibrous roots to form a single, large, shallow, conical or ovoid chamber broadly open to the surface around the plant base. Colonies are highly polygyne and, during the warm season, inhabit multiple nests connected only by above ground trails, over which nests exchange workers. Although monomorphic, worker size may differ significantly between colonies. The colony cycle is dominated by strong seasonal polydomy. From one or two over-wintering nests, the colonies expanded to occupy up to 60 nests by late summer, then retract once more to one or two nests by mid-winter. The worker-to-queen ratio changed greatly during this cycle, with over two thousand workers per queen during fall and winter, dropping to a low of about 300 during midsummer. Most of these summer queens probably die during the fall. Colonies reoccupy roughly the same area year to year even though they contract down to one or two nests in winter. Observation of fights in the contact zone between colonies suggested that the colonies are territorial. The ants subsist by tending aphids and scale insects for honeydew and scavenging for dead insects within their territories.

## Introduction

The life histories of social insect species such as ants span an enormous range of attributes that allow them to exploit an enormous range of terrestrial habitats (Hölldobler and Wilson, 1990). Presumably, the particular attributes of each ant species are what suit it for the particular habitat in which that species is found. The list of attributes of possible importance is both long and uncertain and include colony size, queen number, number of nests, worker size, alate size and number, season of reproduction, the number of matings, mode of colony reproduction, nest location, nest architecture and many more. Unfortunately, there is minimal knowledge of these attributes for the vast majority of ant species, and it is often unclear how known attributes adapt the species to its habitat. Tschinkel (1991) suggested that the collection of such “sociometric” data should be a routine and coordinated activity of ant biologists, and that compilation of such data for many species would reveal informative patterns. Examples of such sociometric studies include the fire ant, *Solenopsis invicta* and the Florida harvester ant, *Pogonomyrmex badius* (Tschinkel 1993; Tschinkel 1998; Tschinkel 1999a; Tschinkel 1999b). Unfortunately, few other such studies have been done and no repository for sociometric data exists.

Of the possible sociometric attributes, queen number and nest number seem particularly fundamental to ant life histories (Keller 1993; Bourke and Franks 1995). The number of queens in a colony of ants profoundly alters several of the key features of colony organization, behavior, and biology. These include mode of founding, territoriality, colony distinctness, colony growth rate, alate size and number, and foraging ecology. Contrasted with monogyny, polygyny is associated with higher population densities, lack of territoriality, low alate and high worker production, low seasonal and lifetime colony size variation, and small workers. The high investment in worker production results in high colony growth rates. For example, the growth rate of the polygyne social form of *Solenopsis invicta* is approximately double that of the monogyne form (compare Tschinkel 1988 with Porter 1991).

Polygyne colonies are often polydomous (Debout 2007), that is, they occupy multiple nests. Polydomy solves the problem created by widely dispersed resources through the establishment of nests close to resources, but it also creates new problems of resource allocation, social regulation, coordination, and communication (Sudd and Franks 1987; McIver 1991; Crozier and Pamilo 1996). Polydomy is not necessarily linked to queen number as polydomous colonies can also be monogyne, as in *Oecophylla longinoda* (Hölldobler 1977), *Leptothorax mayr* (Alloway et al. 1982; Partridge et al. 1997), *Cataglyphis iberica* (Dahbi and Lenoir 1998), *Myrmica punctiventris* (Snyder and Herbers 1991; Banschbach and Herbers 1999; Banschbach et al. 1997), *Cataulacus mcheyi* (Debout et al. 2003), *Camponotus socius* (Hölldobler 1971; Tschinkel 2005) and *Camponotus gigas* (Pfeiffer and Linsenmair 1998).

**Figure 1.**
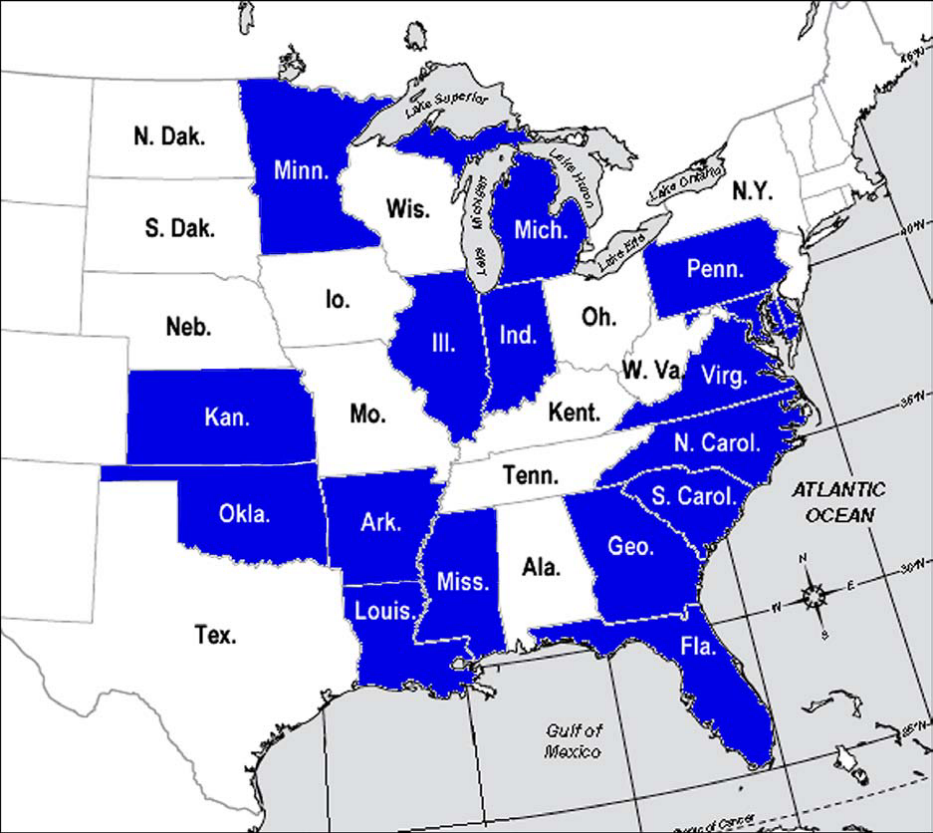
Range map showing the states in which *Dolichoderus mariae* has been reported.

In this paper, some of the basic features of the natural history, seasonality and spatial distribution of the ant *Dolichoderus mariae* Forel, (Hymenoptera: Formicidae) are described. This is not a particularly common ant, but where it occurs, it is conspicuous by virtue of its peculiar nesting habits and locally dense populations. Its geographic range includes most of the eastern and central USA (Figure 1) (Wheeler 1905; Cole 1940; Kannowski 1956; Talbot, 1956; Smith 1979; MacKay 1993; Deyrup 2003). Very little is known of the natural history of this ant. Across its range, the ant excavates its nests under plants with fibrous roots Johnson 1989), including blackberry *Rubus* spp. (Talbot 1956), cattails *Typha* spp (Gregg 1944; Kannowski 1956), and grasses (Wheeler 1905; Cole 1940).

Myrmecologists do not often set out to explore the basic life histories of ant species, choosing instead to focus on particular questions of process or principle with wider implications. Nevertheless, traditional natural history is the well-spring for future ideas and research directions, and such studies require no further justification. Thirty years after a population of *Dolichoderus mariae* was discovered (WRT personal observation) nesting under wiregrass clumps of the coastal plains longleaf pine forests of northern Florida, the population was as vigorous as ever, and the urge to study it finally exceeded the activation energy, resulting in the report below.

## Materials and Methods

### Study sites

**Figure 2.**
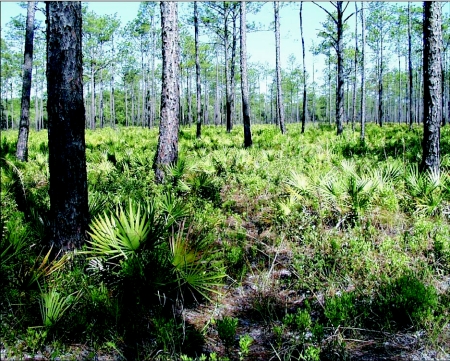
A typical flatwoods site of the type in which *Dolichoderus mariae* may occur. The groundcover of this site in the Apalachicola National Forest in northern Florida consists of palmetto, runner oak, gallberry and wiregrass, while the trees are a monotypic stand of longleaf pine.

The study sites were located in the Apalachicola National Forest, approximately 20 km southwest of Tallahassee, FL. The Forest is divided into numbered management compartments averaging 500 ha in area. In addition to the original population discovered in the early 1970s, a search of 14 areas yielded only 5 additional sites with *D. mariae* populations (Compartments 231, 228, 245, 4 and 13). Two of these (Compartments 228 and 231) were selected for intensive study. The vegetation on these sites consisted of longleaf pine (*Pinus pallustris*) with a mid-story of scattered turkey oak (*Quercus laevis*) and a groundcover of gallberry (*Ilex* spp.), wiregrass (*Aristida stricta*), runner oak (*Quercus* spp.), and saw palmetto (*Serenoa repens*) (Figure 2). The topography is slightly undulating, very sandy soil, with no more than one to two meters of relief, and a water table rarely more than two meters below the surface. Several types of wetlands occupy low areas. *D. mariae* seems to be limited to the higher portions of this landscape.

### The nest and its architecture

The architecture of two live and two abandoned nests under wiregrass clumps was revealed by filling the cavities with a slurry of dental plaster, as described in Tschinkel (2003).

**Figure 3.**
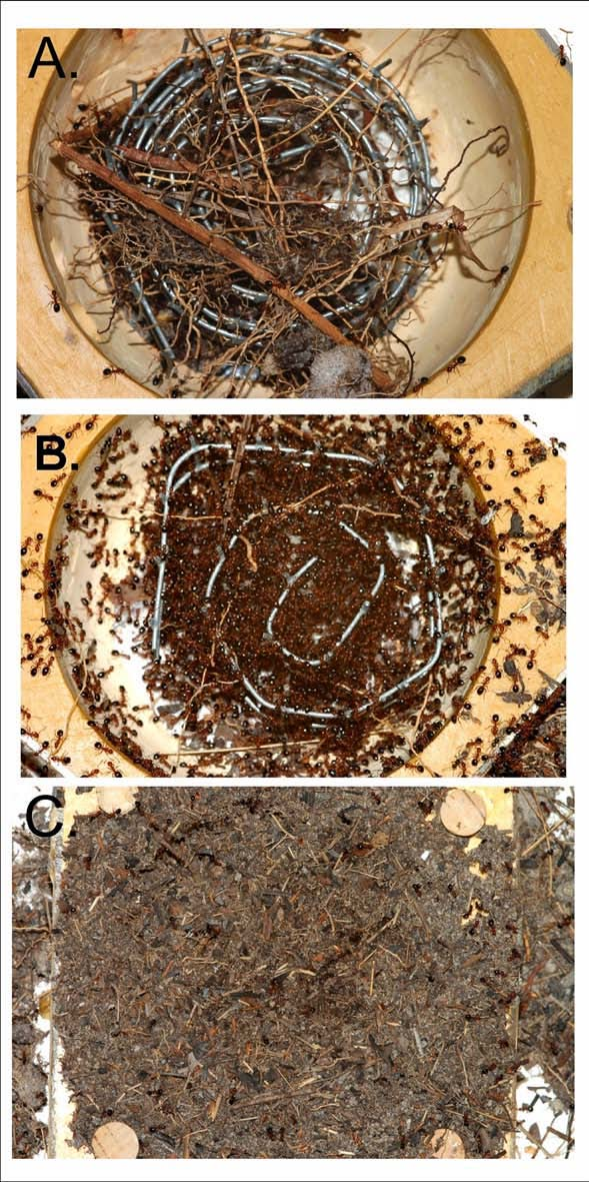
A. An observation nest for *Dolichoderus mariae* consisting of a cavity in plaster contained in a plastic box. Root debris and a strip of hardware cloth serve as a scaffold for the ants. B. The observation nest after the ants have occupied it. The ants arranged themselves and their brood on the scaffolding of wire and root debris. C. After a month or more, the ants have covered the opening of the observation nest with a thatch or felt of chewed plant fiber and debris.

For laboratory observations, nine observation nests were created as a round plaster cavity in a 10.2 cm × 10.2 cm polystyrene plastic box (Seal, 2006) (Figure 3A). In order to mimic the fibrous root structure of the natural nest, a coiled 2.5 cm wide strip of 6.35 mm wire mesh was added to each observation nest along with fibrous root debris from natural nests. Each observation nest was placed in a photo tray with its inner walls coated with fluon to prevent escape, to which the ants were added (Figure 3B). All experimental colonies were housed in the lab at 27° C, fed pieces of beetle larvae, water, and sugar water.

### Queen fecundity

Queens taken directly from the field were placed into test tubes with wet cotton and 0, 5 or 30 workers, and were fed beetle larvae and sugar water. The accumulated eggs were counted daily for 4 days or until egg production ceased. These nests were housed in the lab at 27° C.

To apply these oviposition rates to field conditions, they needed to be adjusted for differences in temperature. Fortunately, the effect of temperature on oviposition rate is similar across ant species at similar latitudes (Porter, 1988; Porter and Tschinkel, 1993). The July field sample needed no adjustment because the laboratory temperature and the mean soil temperature (n=10) were almost identical (27 vs. 26.7° C). In April, the mean soil temperature (n=10 measurements) was 21° C, so the day-one laboratory oviposition rate at 27° C was multiplied by 0.8 to prorate it to 21° C. Field oviposition rates were calculated only for April and July, the only samples in which oviposition occurred. These per-queen rates were multiplied by the number of queens per nest and nests per colony to estimate the per-nest and per-colony egg production rates.

### Census of nests (nest sociometry)

During each season from summer 2005 to spring 2006, three nests (not on the experimental plots) were collected in their entirety by removing a plug of soil 15 cm radius larger than the occupied wiregrass clump. The entire plug containing the host plant and nest was transported back to the laboratory for sorting and counting. Sifting through No. 8 to No. 40 U.S. standard testing sieves separated the live ants from most of the soil and debris. The ants and litter and the total remaining soil were weighed, and then three samples were removed and weighed. Sample weight and the counts of workers, worker pupae, queens, male and female alates, and sexual pupae present in these three samples were used to compute three estimates of the number of each present in the nest. The average of these yielded estimates for each ant type for each nest, and the average of the three nests yielded the estimated seasonal averages.

These seasonal average values were used to compute the corresponding seasonal census values for two entire colonies (231-1 and 228-1) by multiplying the seasonal estimates of each ant type by the number of active nests at each season. Colony size was calculated as the sum of the queens and workers across all active nests of each colony. In addition, the worker birth rates (number of workers per day) were computed as the number of worker pupae per colony divided by the pupal development period. Because the pupal period is strongly dependent on temperature, soil temperatures were measured at the time of sampling. Since ant species at similar latitudes have similar pupal development periods (Porter and Tschinkel 1993) we assumed that the pupal development period of *D. mariae's* southern population was similar to that of the southern population of *S. invicta* (Porter 1988; Tschinkel 1993): 28 days in April at 21° C, 14 days in July at 26.7° C, and 40 days in October at 18°C.

The oviposition rate per colony (number of eggs per day per colony) was calculated as the average number of eggs per nest (adjusted for temperature) times the number of active nests. Oviposition occurred only in April and July. Daily change in colony size was calculated as the colony size on date 2 minus colony size on date 1 divided by the number of days between samples. Positive values indicated size-growth and negative values size-loss. The birth rate for January was zero because egg production ceased below 20° C (Porter, 1988).

The survival from egg to adult was calculated as the worker/colony/day divided by eggs/colony/day.

### Worker size

Five nests from 5 different colonies (not on the experimental plots) were excavated from the field in late May through early July of 2004. The ants were separated from the soil and 50 randomly chosen ants from each of the 5 colonies were dried in an oven for two days at 50° C and individually weighed. The headwidth was then measured using a wedge micrometer (Porter 1983). Head width and body weight distributions were tested for normality using the Kolmogorov-Smirnov and Lilliefors test and skewness.

### Mapping nests and colonies in the field

One month after a prescribed burn, two, 130 m × 60 m survey plots that included many nests were chosen in the upland portion of the long-leaf pine flatwoods of Compartments 228 and 231 of the Apalachicola National Forest. Hereafter the colonies in these plots will be referred to as colonies 231-n and 228-n. Compartment 231 was burned in March 2004 and Compartment 228 in June 2004. All nests of all colonies within each experimental plot were flagged (a different color for each year) and mapped on Cartesian coordinates twice a year (March and June) for two years during the ant's most active season. In addition, the nests of one of these colonies within each plot (231-1 and 228-1) were mapped and inspected for activity during each of the four seasons (April, July, October, and January) for two years. This schedule captured the seasonal changes in the ant's spatial nest distribution, colony size and activity. Note that the seasonal surveys are not a subset of the plot mapping, but took place on different dates.

### Seasonal feeding biology

During each of the four seasons, nests at the base of food sources were recorded, as were the plants on which the ants tended aphids, revealing seasonal feeding patterns.

### Establishing colony limits

Two criteria were used for establishing colony identity. First, nests connected by active trails, and therefore exchanging workers, were assumed to belong to the same colony. Nests not thus connected were subjected to aggression assays in which workers from candidate nests were placed in a common arena. Aggression or fighting between them indicated that the workers originated in different colonies. Aggression was rated using the scale of Giraud et al (2002), from least to most aggressive: 0 (no response), 1 (antennation), 2 (avoidance), 3 (gaster raising), 4 (leg pinning), and 5 (fighting: biting or gaster flipping). When aggression occurred, it was usually 4 or greater on this scale. Those nests that were not connected by trails yet showed no aggression towards each other were assumed to be from the same colony.

## Results

### Overview

It became apparent that *D. mariae* exists in distinct, polydomous colonies that expand and contract with the seasons. Because the colony, not the nest, is the probable functional unit, it was necessary to establish which nests were included in each colony, and then characterize the size, composition and seasonal changes in these colonies. To accomplish this all nests in the experimental plots were mapped and then, by means of connecting trails and aggression assays, the nests constituting each colony were determined. Randomly chosen nests (not on our plots) were selected during each season for census of the ants within them. These census values were then used to estimate (and the estimates are admittedly rough) the seasonal changes in the composition of entire colonies on the plots.

### The nest

#### The nest and its use by the ants

**Figure 4.**
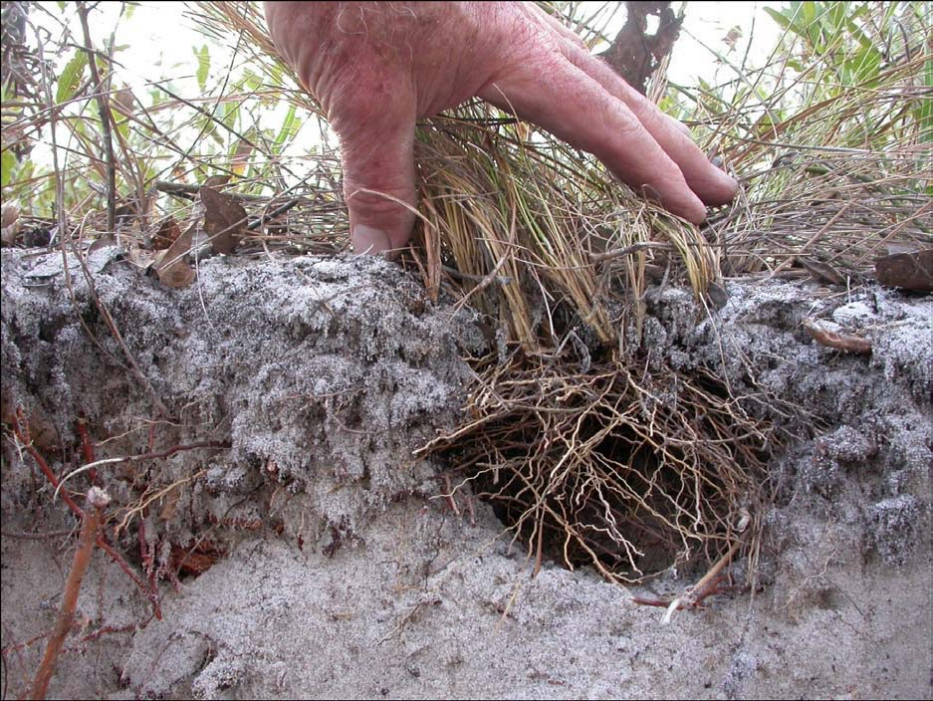
A section through an abandoned nest of *Dolichoderus mariae* showing the roots that traverse the chamber and serve as a scaffold for the ants. Chambers never extend beyond the roots of the plant under which they are excavated. Once abandoned, these chambers often serve as a refuge for a variety of vertebrate and invertebrate animals.

**Figure 5.**
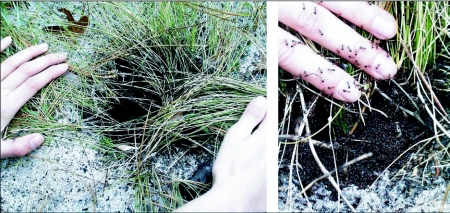
(Left) An occupied nest of *Dolichoderus mariae* showing the mass of ants that form a mat at the chamber opening around the base of the wiregrass clump. (Right) Detail of same. The ants are rather placid and not easily panicked.

When the ants excavate the soil beneath wiregrass clumps, they expose the fibrous roots intact inside the chamber, providing scaffolding (Figure 4) on which the ants arrange themselves, their queens and their brood. The observer is thus presented with a cavity packed full of workers (brood is not easily visible), with a layer of workers exposed at the surface surrounding the wiregrass clump (Figure 5). The ants are slow moving and not easily perturbed, and it is possible to insert one's finger deep into the mass of ants without panicking them. The massing of ants around the base of the wiregrass is not a basking behavior, for the same behavior was observed on overcast winter days, with temperatures hovering just above freezing.

**Figure 6.**
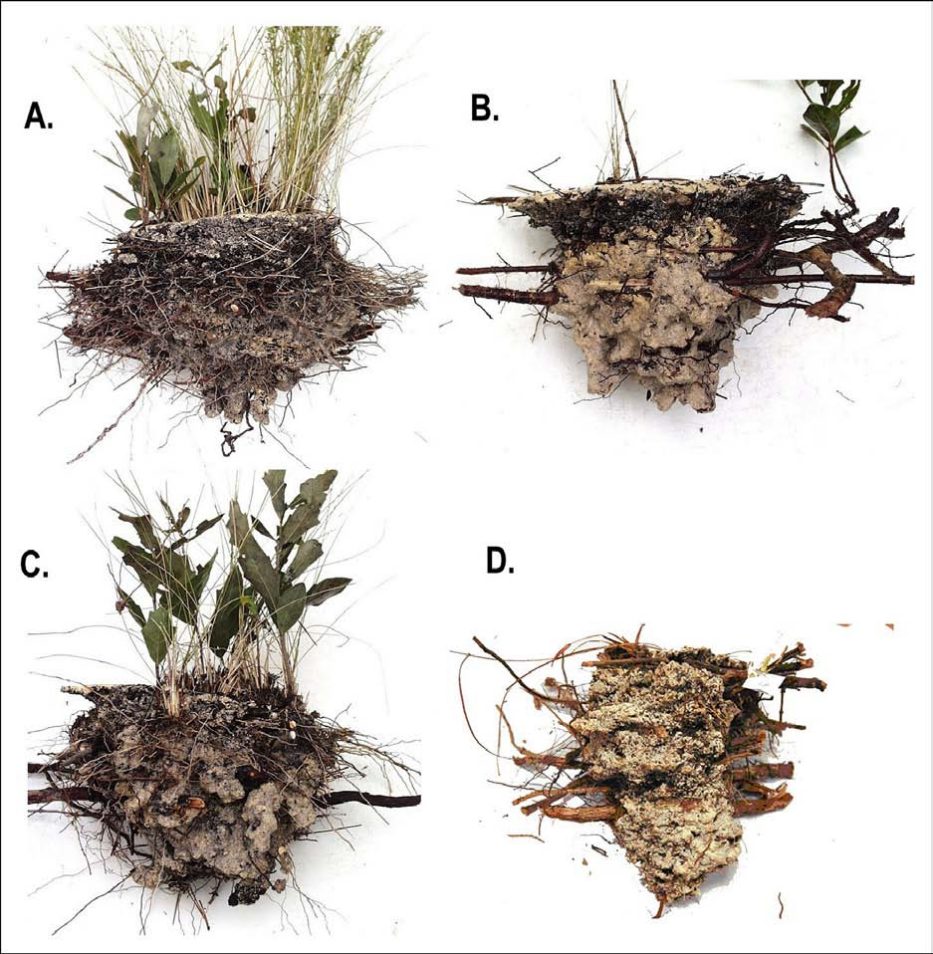
(Top) Casts of the nests of two *Dolichoderus mariae* nests, showing the simple nature of the nest cavity. Both nests were occupied. (Bottom) Casts of two nests that had been abandoned.

The dental plaster casts revealed that these *D. mariae* nests consist of a shallow, single, large, conical chamber beneath the wiregrass (Figure 6A–D). Nests averaged 15 ± 4 cm (mean ± SD) in depth and 930 ± 350 cm3 in volume. No tunnels emanated from the nests. The walls of the chamber never surpassed the wiregrass root system so larger nests were associated with larger wiregrass clumps. Chambers retained their conical shape even after abandonment (Figure 6C,D). However, the plaster casts of abandoned nests appeared to be pitted while the plaster casts from active nests were smooth, suggesting that some agent of degradation, such as scavengers or other soil arthropods, had been active.

**Figure 7.**
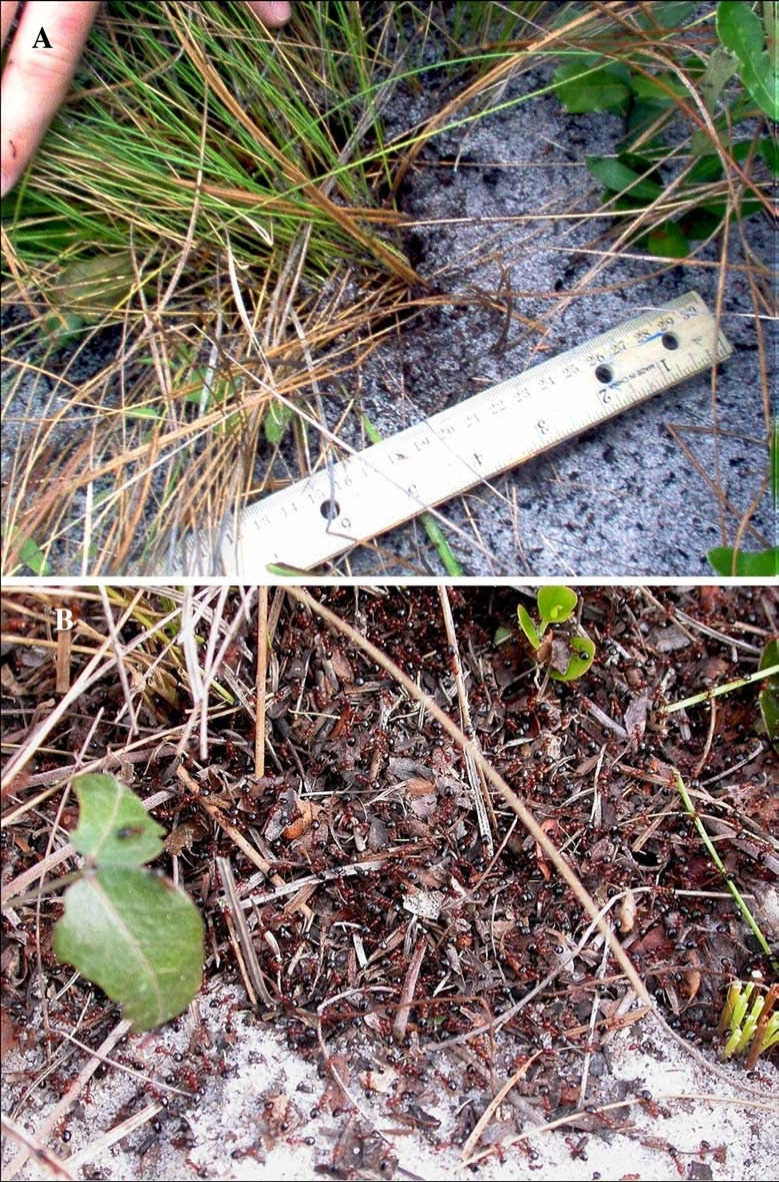
As in the laboratory, some field nests of *Dolichoderus mariae* cover their nest openings with chewed fiber and debris. (Top) An example of chewed fiber. (Bottom) An example composed mostly of debris.

When provided with artificial nests that mimicked this root scaffolding, the ants arranged themselves in a generally similar manner to that seen in natural nests. Thus, in all nine experimental nests, the ants used the wire mesh and debris as scaffolding for their brood, workers, queens, and alates (Figure 3B). After about a month, four laboratory nests covered the top opening with a thatch of chewed bits of plant material to produce a felt or thatch of paper consistency (Figure 3C). In the field, as the ground cover regrew during the second post-fire year, such thatch was also seen on 4 of 10 nests during April 2005 in colony 231-1 and 11 of 19 nests in colony 228-1. Nests with a thatch were observed during all seasons (Figure 7). It was not clear why some nests produced thatch and others did not.

### Nest sociometry through the seasons

**Figure 8.**
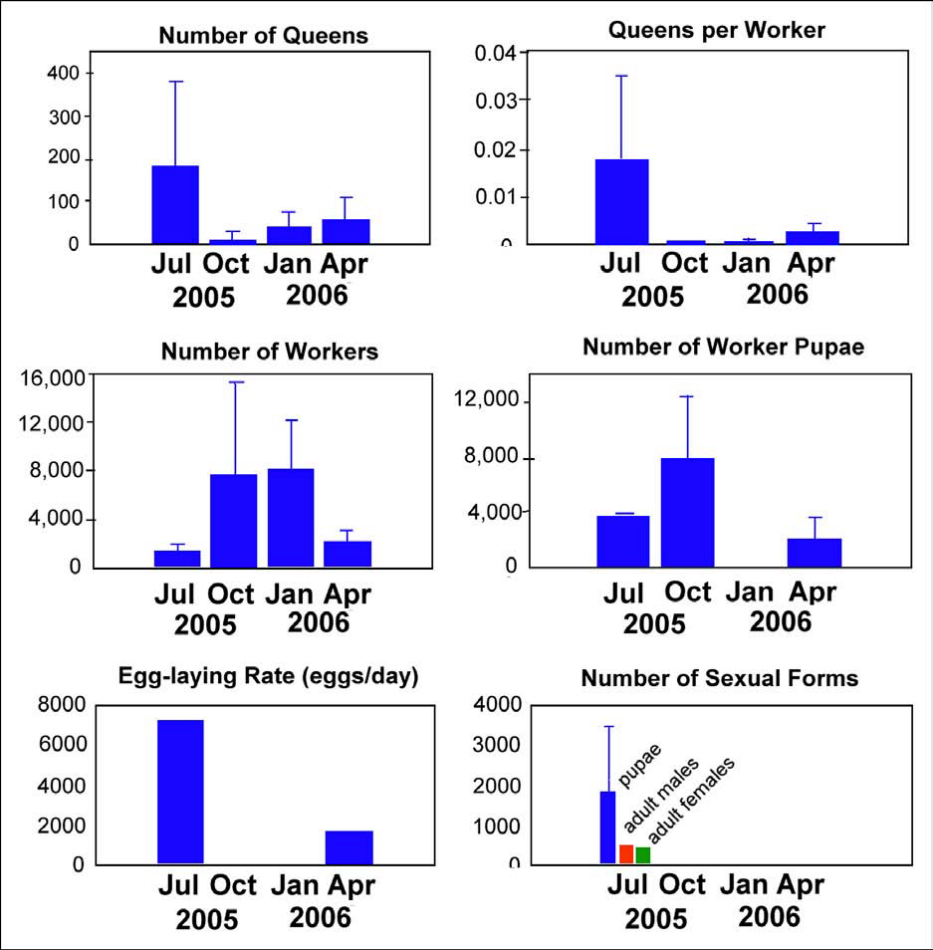
Census values for seasonal nest samples of *Dolichoderus mariae*. Each statistic was derived from three samples taken from three nests.

The three nests excavated during each season yielded the mean counts of queens, workers, worker pupae, sexual pupae, male and female alates in the average nest during each season (Figure 8). Variation of most of these counts among nests was generally high (the following statistics are all from one-way ANOVA). The average nest-unit contained the most workers October through January (73,000 to 76,000) and the fewest April through July (13,000 to 19,000) (F_3,25_ = 3.35; p< 0.05). The smaller size during the most active season was probably the result of nest fissioning or budding. Queen number followed the opposite pattern, with significantly lower numbers (12 to 59) October through April than in July (180 per nest) (F_3,25_ = 3.844; p< 0.05). This increase during summer was probably the result of the adoption of newly mated queens produced during the reproductive season and retained in the nest after the nuptial flights, although mating in the nest cannot be ruled out. As a result of the opposite trends in worker and queen numbers, queen/worker ratios were 2 to 10 times as high in July (i.e. during peak reproductive season) as October through April (F_3,25_ = 7.757; p< 0.001). Each July queen was accompanied by 290 workers, whereas each January queen was associated with 2400 workers (F_3,25_ = 4.675; p< 0.05). July and October were intermediate in numbers of workers associated with the queen.

### Queen fecundity

The initial (day 1) oviposition rate of queens was similar no matter how many workers accompanied them, means ranged from 27 to 40 (SD 14 to 25; F_2,41_ = 0.528; n.s.), but continued egg production depended upon the number of workers. The overall mean for day-one was 33.3 eggs/day (this value was prorated for temperature and used to estimate egg production in the field). On day 2, queens without workers or with 5 workers averaged only 1 and 8 eggs respectively, significantly fewer than those with 30 workers that laid 22.4 eggs (F 2,41 = 10.7; p< 0.0005). On day 3, egg production of queens with 30 workers was still significantly higher (15.4 vs. 0 and 0.9) than those without workers or with 5 workers (F _2,41_ = 13.8; p< 0.00005). By day 4 all queens had ceased laying eggs. These results not only give estimates of queen oviposition rates, but also suggest that the maintenance of oviposition requires input from large numbers of workers.

**Figure 9.**
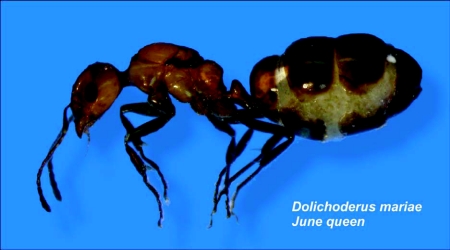
A physogastric *Dolichoderus mariae* queen typical of those in June. During the summer, a nest may contain hundreds of such queens.

**Figure 10.**
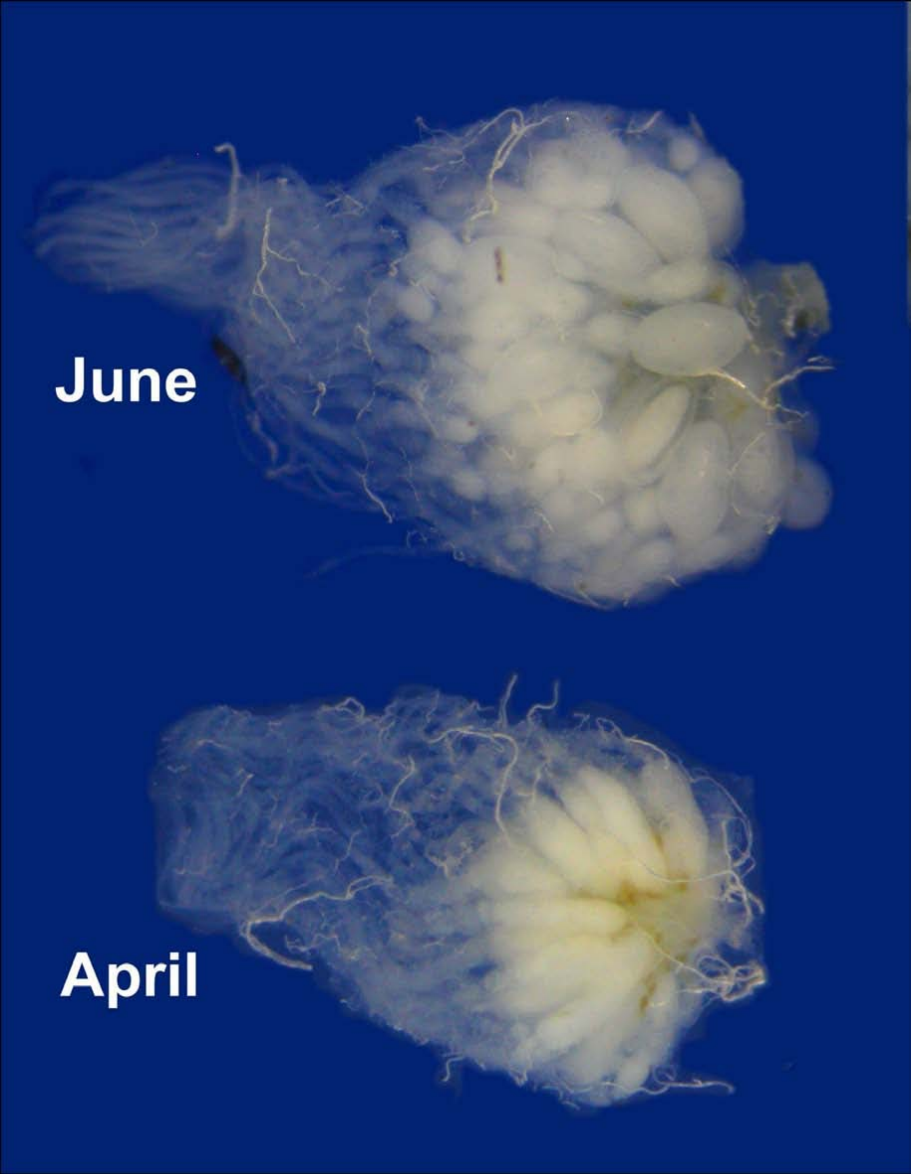
The ovaries of *Dolichoderus mariae* queens in June and April, showing the greater number of ovulation-ready terminal oocytes in June. Ovaries contain about 50 ovarioles, but rarely have more than 2–3 vitellogenic ooctyes per ovariole.

Dissections of queens collected in January, April and June of 2006 revealed the anatomical basis of their reproductive output. Queen ovaries were composed of an average of about 50 ± 3.6 ovarioles each. The large, double-lobed spermatheca of most queens contained sperm, indicating that they were mated. However, in one of the two June nests, 4 of the 8 queens were unmated, yet their ovaries were as developed as those of mated queens. In January, ovaries were inactive and the queens showed little sign of physogastry. By April, queens were visibly physogastric and their ovaries contained a total of about 70 ± 24 vitellogenic oocytes, of which about 12 ± 12 were of a size large enough to be ovulated. By June, all queens were physogastric (Figure 9); practically all ovarioles contained an oocyte large enough for imminent ovulation. Most queens bore about 90 of these, and a total of about 152 ± 41 vitellogenic oocytes. Figure 10 compares the ovary of an April queen with that of a June queen, showing that the main difference is that there are more ovulation-ready oocytes in June. In all samples, ovarioles with more than three vitellogenic follicles were rare, and most ovarioles had only one or two. Neither in April nor in June was there any evidence of strongly skewed oviposition rates among the queens, confirming similar observations from oviposition in the laboratory. Combining the oocyte counts with the oviposition rates (90 and 33, respectively) suggested that queens lay about a third of their terminal oocytes daily.

Combining the laboratory-determined egg laying rate of individual queens (mean 33.3 eggs/day) with the mean number of queens per nest yielded the (temperature-adjusted) estimate of daily production of eggs per nest in the average nest for each season (Figure 8). Egg laying occurred only in April and July with the July rate (6000 per day per nest) almost quadruple the April rate of 1500. However, because of high variation and small sample size, these differences at the nest level were not significant.

Egg survival appeared to be very low. Only 3 to 5 % of the eggs survived to adulthood in April and July. The fate of 95 to 97% of the eggs needs further investigation. Perhaps many of the eggs were fed to larvae or destroyed by queens in competition with each other.

### Worker birth rates

Worker pupal census is of interest because it can provide an estimate of worker birth rates when the census is divided by the pupal development time in days. Worker pupae were almost absent in January. After egg laying commenced in the spring, worker pupae increased in abundance through April and July, and peaked in October (F_3,25_ = 12.55; p< 0.00005), lagging the July peak in egg laying rate, and coinciding with a drastic decline in queen numbers. Nevertheless, after adjustment for the ambient temperature, these October peak worker pupae censuses resulted in lower worker birth rates than in July.

### Alates

Sexual larvae first appeared in low numbers (23 per nest) in April and reached a maximum of 1800 in July (F_3,25_ = 9.991; p< 0.005). Sexual adults were essentially present only in the July sample, with a mean of 480 males and 380 females per nest (but again, variation was high). The large number of sexual larvae still present in July suggests that the mating season was far from over in July, possibly continuing at least through August. However, by January, no sexuals, eggs, or brood of any type were present in any of the nests.

### Worker size and size variation

**Figure 11.**
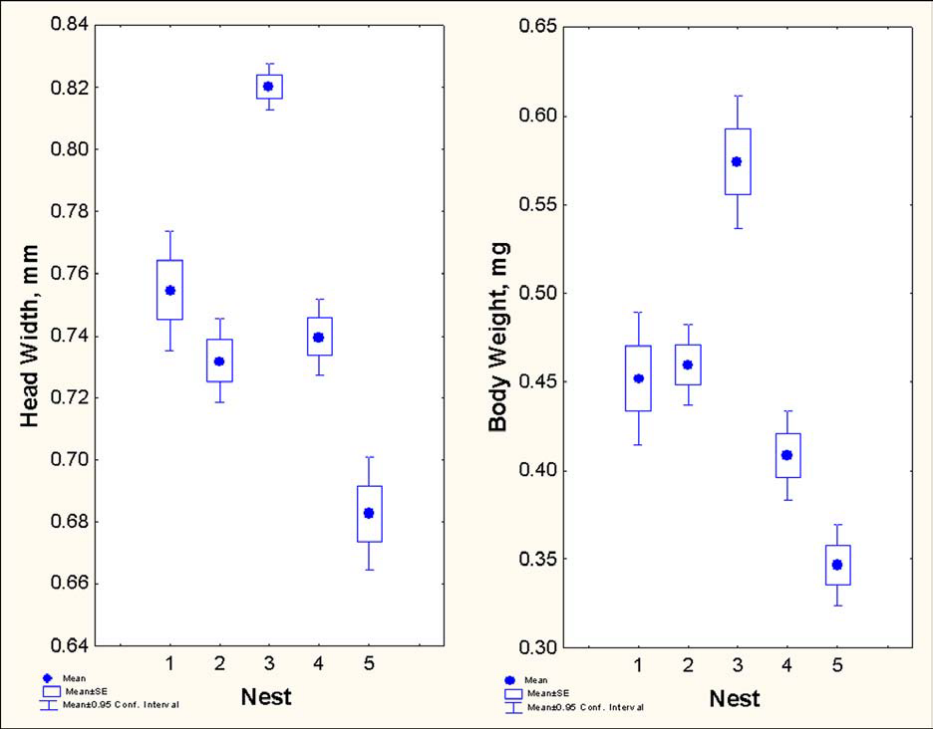
*Dolichoderus mariae* worker headwidth (Left) and body weight (Right) vary significantly among colonies, but within colonies, worker size is monomorphic and varies only modestly.

**Figure 12.**
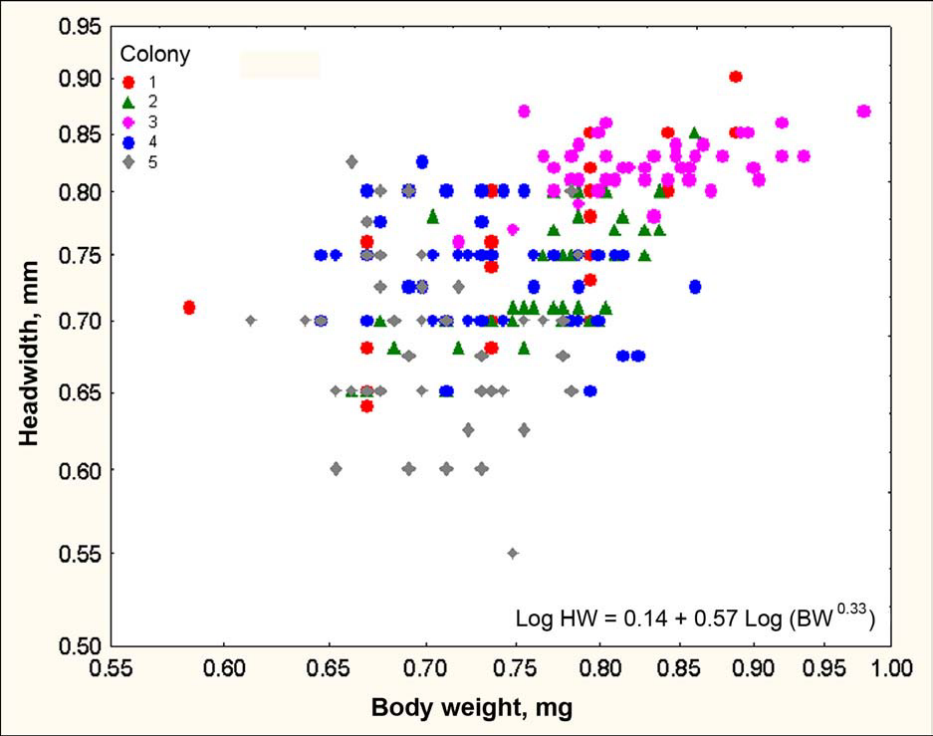
Across *Dolichoderus mariae* colonies, headwidth is significantly related to body weight, but within most colonies, this relationship is not significant. Body weight in such colonies is related to something other than body dimensions.

*D. mariae* workers are monomorphic. Although workers from different colonies differed significantly in headwidth and body weight (Figure 11) (headwidth: F _4,232_ = 43.4; p< 0.000001; weight: F _4,232_ = 30.5; p < 0.00001), variation of worker size within colonies was small;the coefficient of variation for headwidth was 3 to 9%, and that for body weight 16 to 29%. The mean head width ranged from 0.73 mm to 0.82 mm across the five nests sampled, and the mean body weight ranged from 0.35 mg to 0.57 mg (Figure 11). A regression of the log cube root of body weight on the log of the head width (a standard allometric plot) found a significantly positive slope (about 0.7) for colonies 1 and 2, but the remaining three slopes were not different from zero (Figure 12). The absence of a relationship between head dimension and body weight in three of the five colonies suggests that much of the variation in body weight may result from variation of body fat or food stored in the gaster. Colonies 1 and 2 contained larger workers that were more variable in size, possibly accounting for the significant overall relationship between head width and body weight. However, the colony mean of body weight was strongly related to the colony mean of headwidth, suggesting that across colonies, larger workers were heavier. The reasons for the variation of worker size among colonies are not known, but could be related to nutritional status, nest size or queen number.

### Mating flights

Mating flights occurred in the early morning between 7:00 a.m. and 8:30 a.m the day after heavy rains. In 2004 and 2006 mating flights were seen in July and in 2005 they were seen in late May and June. Rains came later in 2004 and 2006 than in 2005, possibly accounting for this difference in timing. Thousands of male alates left their nest and entered other nests. Meanwhile, very few (∼50) female alates flew away, the majority of female alates remained in the nest, probably mating in or on the nest. By 9:00 a.m. all mating flights had ceased.

### The colony

#### Seasonal patterns at the colony level

**Figure 13.**
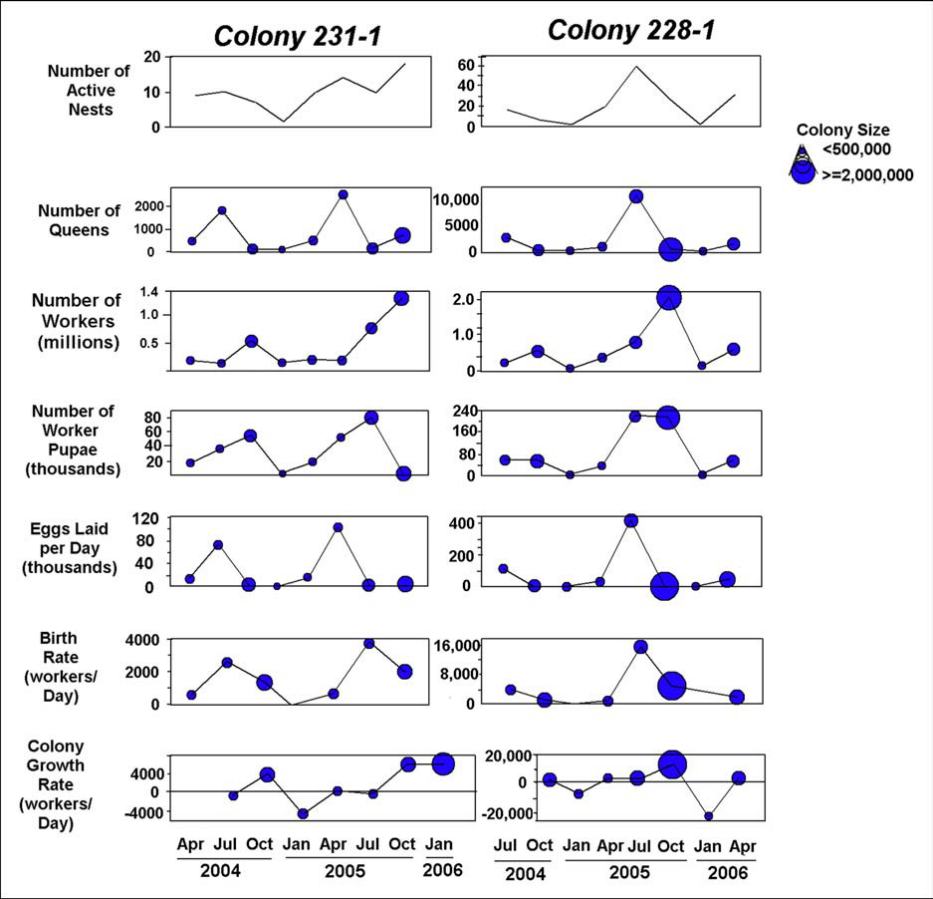
Colony census and life-history attributes of *Dolichoderus mariae* through the seasons. These two colonies were surveyed four times a year, and the census values computed from the nest excavation/census data in Figure 8 (see text). Colony size is usually lowest in winter, but colony 231-1 continued to grow during January 2006, possibly because of warm weather and abundant homopterans. The size of the closed circle indicates colony size (number of workers).

The number of nests in each colony changed with the seasons, acting as the main driver of changes in our colony size estimates. Because the colony is the functional unit of *D. mariae*, the census calculations at the colony level presented below are more biologically meaningful than the nest census means. As it was assumed that the nest census data would be similar for all years, the census values were multiplied by the number of active nests in each colony to derive the corresponding census values for colony 231-1 and colony 228-1 for both years (Figure 13). The results present an approximate picture of the seasonal lives of colonies of *D. mariae*.

The two colonies differed greatly in size, and each increased greatly in size over the two-year period. Because their patterns were somewhat different, they are described separately. Recall that most of the reported values below are extrapolated estimates. In both colonies, spacing between nests varied from centimeters to meters.

Colony 228-1 showed a strong seasonal pattern in the number of active nests, peaking at 16 nests containing an estimated 2900 queens in July 2005 and 59 nests with an estimated 10,700 queens in 2007. Queens began to lay eggs no later than April, resulting in growth of the colony until at least October. During the summer peak in 2005, colony 228-1 produced an estimated 96,000 eggs and 2800 new workers daily, and in 2006, 360,000 eggs and 10,500 new workers daily. These production rates brought the colony to its annual maximum of about half a million workers by October 2005, and about 2 million in 2007. After summer, all colony size measures shrank to their minima in January. By January 2005, colony 228-1 had contracted from July's 16 nests to a single nest with about 40 queens. Of the half-million workers, 84% had died, leaving 82,000 surviving workers. By January 2006, the 59 nests present in July had contracted to two nests with 90 queens. Of the 2 million workers alive during July, about 90% had died, leaving 160,000 surviving workers. Between the annual minimum and maximum, colony size thus changed 6 to 12 fold, and the number of active nests up to 30-fold. Comparing the peak size in year 1 with that in year 2, colony size increased 3 to 4--fold in the number of nests, workers and queens.

Colony 231-1 followed a less dramatic pattern. By July 2005, it peaked at 10 nests containing an estimated 1800 queens, and by July 2006, at 14 nests with 2500 queens. As in colony 228-1, queens began to lay eggs no later than April, but the colony did not grow very steadily; there were actually fewer nests in October than in July (10 vs. 7). Nevertheless, worker number peaked at an estimated 530,000 in the October 2005. During the summer peak in 2005, colony 231-1 produced 60,000 eggs and 1800 new workers daily, and in 2006, 84,000 eggs and 2500 new workers daily. These production rates were much lower than colony 228-1, and resulted in a considerably lower growth rate of colony 231-1. During 2005, all colony size measures shrank by 80% to their minima in January; 2 nests containing about 160,000 workers and 40 queens. January 2006 was anomalous when compared to the two years of colony 228-1 and to less-intensely studied colonies. Rather than declining in size after October, colony 231-1 continued to grow, reaching a size of 14 nests with an estimated 1.5 million workers and 800 queens by January of 2006, the last sample of our study, and a 9-fold increase over the previous year. Homopterans remained abundant in January 2006, suggesting that at least some of the changes in colony size were driven by food availability. From July 2005 to July 2006, the colony only increased 40% in size, much less than the 3-4-fold increase of colony 228-1. The origin of these differences is obscure.

Finally, it should be noted that a large fraction of each colony was on the surface foraging or traveling between nests, rather than in the nests censused; therefore, colony size is probably much larger than was estimated.

**Figure 15.**
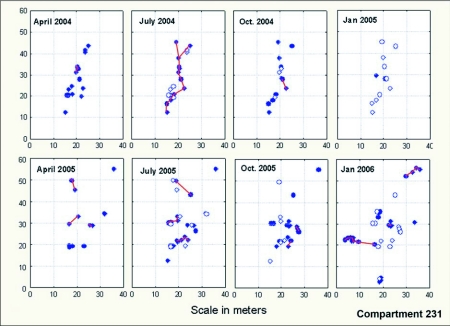
Maps of the nests of colony 231-1 through this study. Closed symbols indicate a nest occupied by *Dolichoderus mariae* during that survey, whereas open symbols indicate nests occupied in the previous survey but abandoned in the current one. Red lines show the connecting trails between nests. Connectedness was highest in mid-summer.

**Figure 16.**
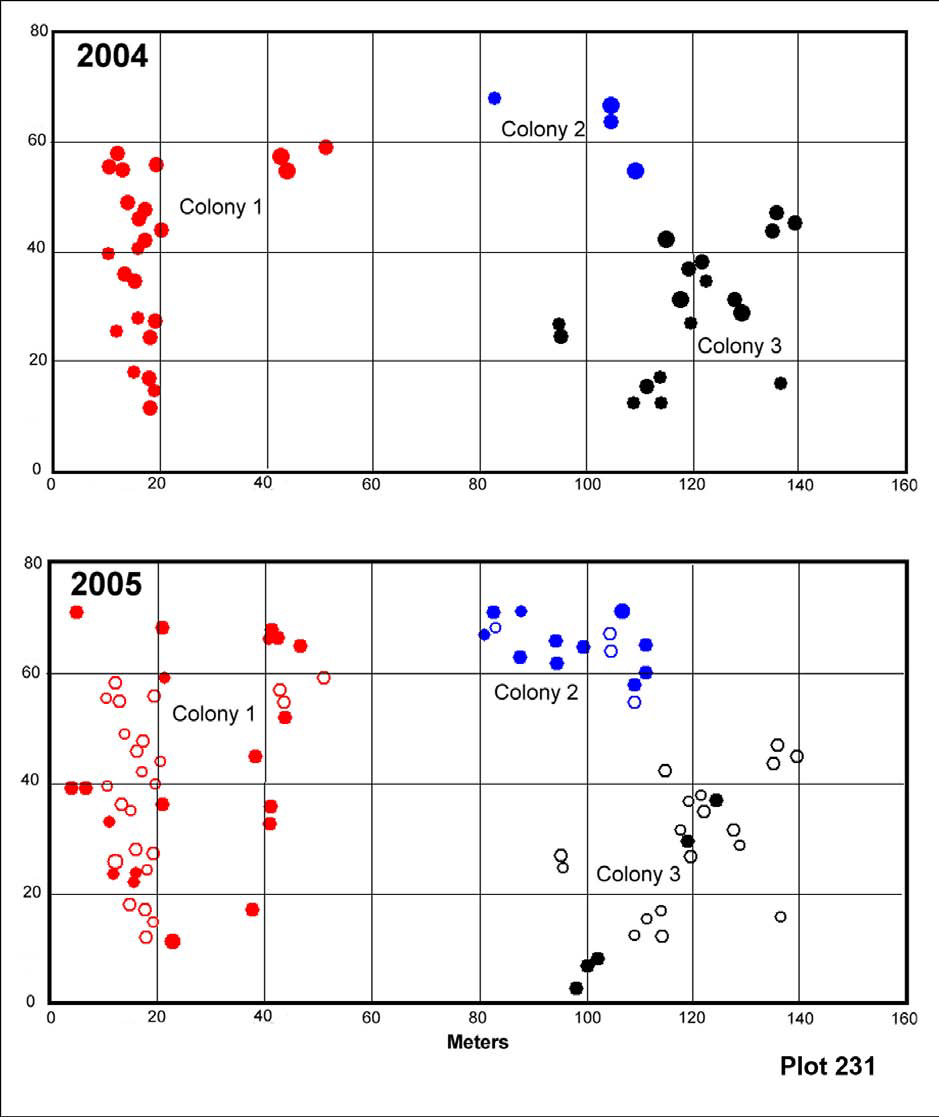
Plot 231 in 2 successive years. The plot contained three different *Dolichoderus mariae* colonies that, although shrinking to only one or two occupied nests in January, reoccupied the same area in the following year. The zones between these colonies were the sites of occasional fights. Closed symbols indicate a nest occupied during that survey, whereas open symbols indicate nests occupied in the previous survey but abandoned in the current one. Colony 231-1 is the same as in Figure 15, but the survey dates are not identical.

Figures 15 and 16 provide maps of these two colonies, showing the expansion and contraction in space and time, and the trails connecting nests within the colonies. As already noted, colonies had the lowest number of active nests during the winter and the highest number in July, decreasing again to the winter minimum thereafter. The degree of connectedness by means of trails between nests paralleled the number of active nests; highest in July and the lowest in January. This paralleled the availability of homopterans to be exploited for food.

### Territoriality, space and movement

**Figure 17.**
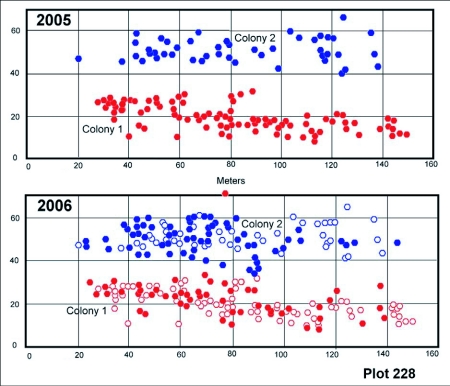
Plot 228 in successive years. The plot contained two different *Dolichoderus mariae* colonies that, although shrinking to only one or two occupied nests in January, reoccupied the same area in the following year. The zones between these colonies were the sites of occasional fights. Closed symbols indicate a nest occupied during that survey, whereas open symbols indicate nests occupied in the previous survey but abandoned in the current one. Some of the nests are too close together to be shown as separate symbols, hence the number of symbols does not agree with the numbers cited in the text. Colony 228-1 is the same as in Figure 14, but the survey dates are not identical.

*D. mariae* populations are extremely local, but where populations do occur, the ants carpet the landscape, and several colonies occur in close proximity. Nests from the same colony were always contiguous, and nests from different colonies were never intermixed. Colony size and number varied within and between the survey plots. During the period of maximum nest number (May through July), the 7800 m^2^ plot in compartment 231 contained three colonies (Figure 16). These consisted of 25, 4 and 18 nests in 2004, and 22, 12 and 5, respectively, in 2005. The plot in compartment 228 contained two colonies, one of which had 108 nests in 2005 and 57 in 2006, while the other had 53 nests and 63, respectively (Figure 17). Clearly, colonies may grow, remain constant or decrease from one year to the next. The reasons for this variation are not obvious, but may be related to the sporadic occurrence of homopterans in their territories.

**Figure 18.**
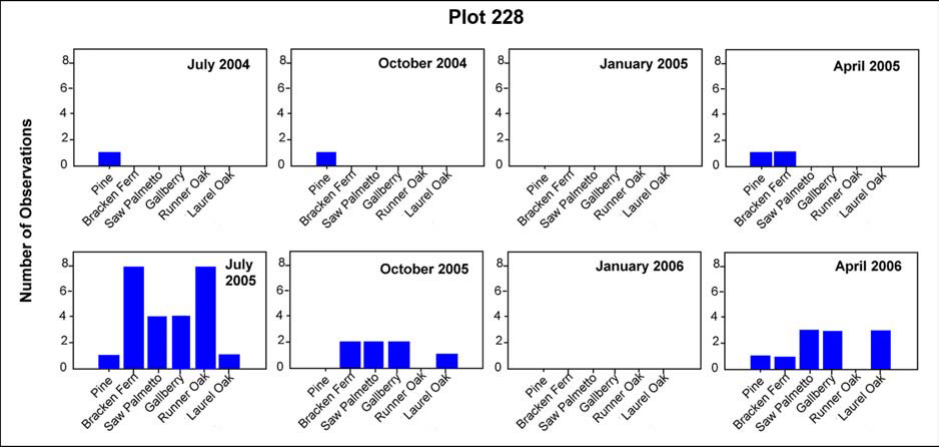
Exploitation of homopterans by *Dolichoderus mariae* on various food plants on plot 228, shown as the number of observations on each plant during each survey.

In spite of contracting to one or two nests during the winter, each colony reoccupied more or less the same area each year (Figures 17, 18). A test comparing the mean nest coordinates between years showed that neither colony 228-1 nor 231-1, as a whole, moved its average location (F16,1172 = 0.96; n.s.), although nests may shift within colonies. This pattern suggests territoriality, that is, the stable occupation of a piece of ground defended against neighbors. Indeed, when workers from different colonies were mixed, they fought, suggesting that the colonies defend an absolute territory by means of aggression. Colonies were usually separated by a zone in which neither nested (Figures 17, 18). When foragers from different colonies came into contact, territorial battles ensued, confirming the existence of territoriality.

### Nest-budding

In April and July, new nest establishment through budding was observed. Workers, queens, and brood moved on connecting trails between nests. Especially between April and July, the period with the highest rates of new nest establishment, queens were observed traveling the connecting trails along with workers. Nests were also observed being disbanded through reverse budding (returning to a larger nest) in October.

### Seasonal feeding biology

**Figure 19.**
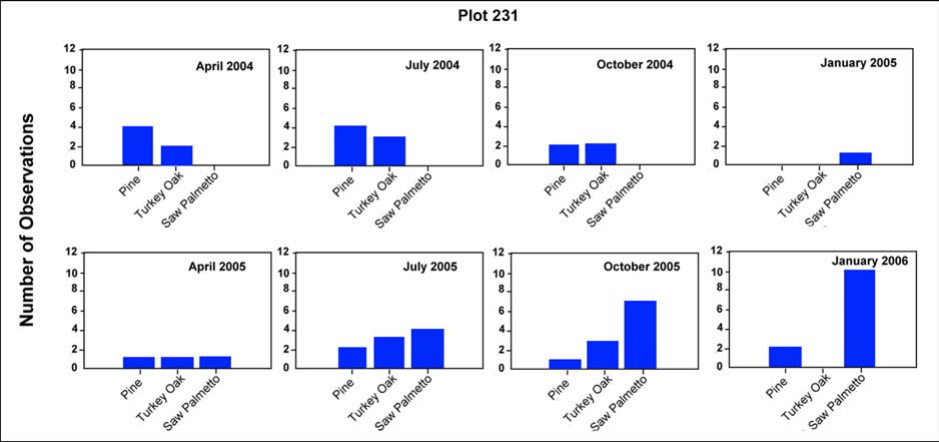
Exploitation of homopterans by *Dolichoderus mariae* on various food plants on plot 231, shown as the number of observations on each plant during each survey.

**Figure 20.**
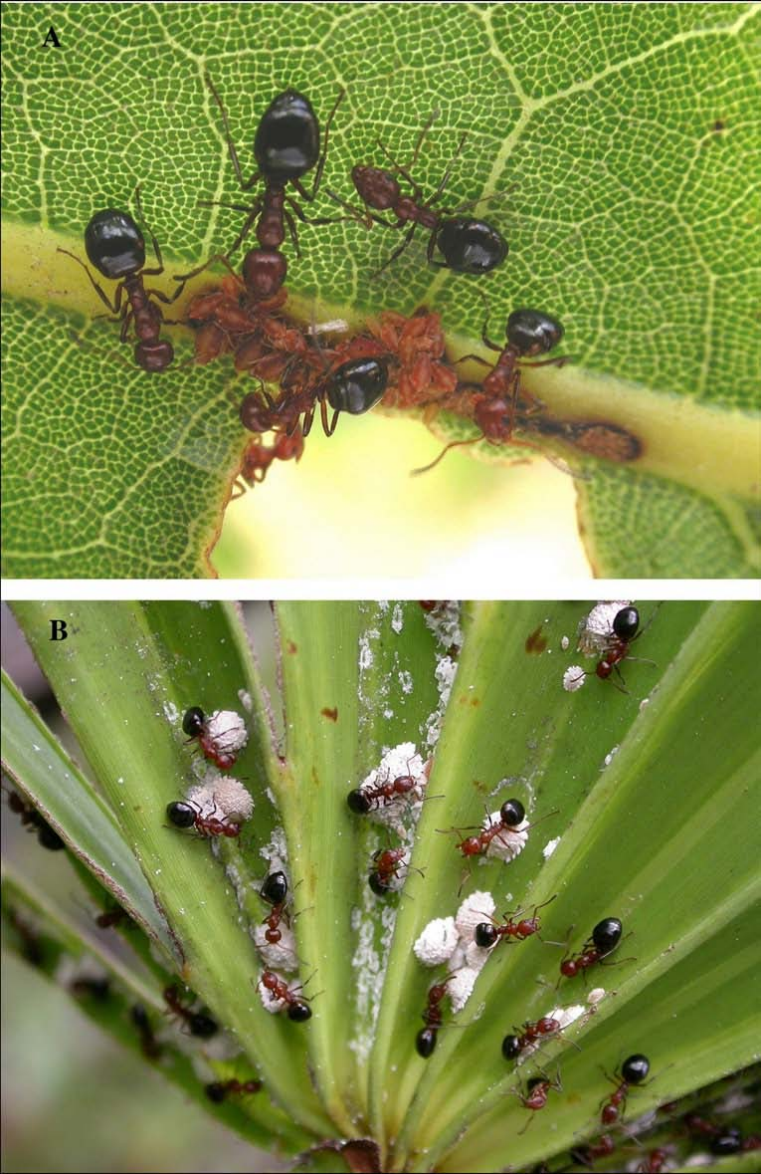
(Top) *Dolichoderus mariae* workers tending aphids on turkey oak. (Bottom) *D. mariae* workers tending mealybugs on palmetto.

Figures 18 and 19 shows the variation through the year of the number of nests at the base of plants that harbored aphid and scale insects that the ants visited to collect food, probably primarily honeydew, but possibly the insects themselves as well. Disturbance of such small satellite nests often resulted in the workers evacuating brood back to a larger nest. The particular homopteran species tended and the plants harboring them varied with season (Figure 20 shows one example). Over the two-year observation period colonies 228-1- and 231-1 tended colonies of homopterans underneath the bark of long-leaf pine trees, on bracken ferns, saw palmettos, gallberry shrubs, runner oak, and laurel oak trees. Aphid colonies typically contained thousands of individuals, while scale colonies (e.g. Pseudococcids) contained hundreds. Ants returning from tending homopterans had extended gasters, suggesting that the ants' crops were full of honeydew.

**Figure 21.**
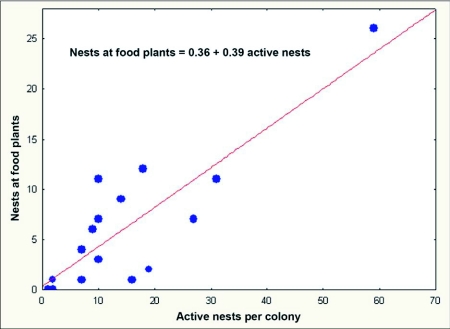
An average of about 40% of all active *Dolichoderus mariae* nests were located at the base of a plant harboring homopterans being exploitated by the ants. Dispersal to exploit such food opportunities may be a major stimulus for nest fission in this ant.

Colony 231-1 visited homopterans on pine and turkey oak during 2004, but beginning in January 2005, homopterans on saw palmetto became increasingly important, dominating as a food source after July of 2005. In January 2006, homopterans were still abundant on saw palmetto, possibly accounting for the lack of contraction of this colony during winter. Colony 228-1 also visited colonies on pine in 2004, but by July of 2005 and again in 2006, the colony was visiting abundant homopteran colonies on four or five different species of plants, with pine the least visited. This abundance coincided with a large increase in the size of this colony. There is a strong correlation between the number of active nests and the number of nests at the base of food plants (Figure 21; regression: nests at food plants = 0.36 + 0.39 active nests (F_1,14_= 36.45, R^2^ = 70%; p<0.00005); in other words, about 40% of the nests are associated with food plants. It is likely that the fortunes of *D. mariae* depend on finding sufficient homopterans. The ant is probably opportunistic with respect to the species of homopterans it exploits, and it is also possible that the winter contraction is facultative, failing to occur when food remains abundant during winter (as in colony 231-1).

## Discussion

The typical colony cycle of *D. mariae* was dominated by strong seasonal polydomy, beginning in January with one or two nests. Colony size and queen number were near their minima between January and April. Egg production commenced sometime between January and April so that by April, worker birth rate began its climb toward its maximum in July. The increase in the worker and queen population drove the increase in the number of nests through budding, which was accompanied by a decrease in the number of workers in each nest as fractionation proceeded (Figure 8). Colony growth could continue until at least October. As winter approached, a large die-off of both queens and workers occurred, along with reaggregation into fewer nests, so that the over-wintering colony contained only a small percentage of the summer's workers and queens, who were then found in one or two nests. Clearly, the great majority of both workers and queens lived considerably less than a year.

The spring-summer expansion represents an enormous growth rate; the worker force increased up to 25-fold in 9 months (doubling every 6 weeks), and the number of queens increased 250-fold in 6 months. The number of nests increased up to 60-fold in 6 months as the colony reoccupied its territory. However, these patterns may differ for different colonies. Growth may continue into January, and the rate and amount of growth can differ greatly. Moreover, comparing mid-summer sizes, colonies may grow enormously from one year to the next, they may remain the same size, or they may shrink. It seems possible that much of this variation is based upon the vagaries of the capricious blooming and fading of homopteran populations, and whether these are present within the ants' territories (see below).

The annual cycle in the queen population can be even more dramatic than that of the worker population, differing up to 250-fold between summer and winter. In summer, there may be thousands of queens in a colony, pumping out hundreds of thousands of eggs every day. At least early in the year, many of these eggs must develop into sexuals, because by July the number of workers per queen decreased to as little as 3% of its January value. In July, the average nest contained 25% sexual forms by weight. Once mating flights commence, queen number probably increased rapidly in the nests, and these queens must increasingly produce workers, rather than sexuals. The fact that mating occurs in or on the nest suggests that relatedness among queens and workers is high.

Queens begin to die off rapidly sometime between July and October, so that by October the number of queens had returned to near its midwinter value. Oviposition ceased before October, but worker pupae were still present. These patterns suggest that the summer and overwintering queens were functionally different. Clearly the great majority of queens and possibly all queens live less than a year, but it is also possible that over-wintering queens live multiple years. Queens alive in the spring produced most, or possibly all, of the sexuals, whereas summer queens probably mostly boosted colony size rapidly by producing workers. This cycle has similarities to that of many ant species, producing sexuals in the spring, and mostly workers thereafter (Hölldobler and Wilson 1990).

Although the life cycle of *D. mariae* appears to be a simple seasonal one, some evidence suggests that season itself may not be the direct driver. For example, the large size of colony 231-1 in January 2006 was associated with large populations of homopterans as well as warm weather, but not with photoperiod. Clearly high winter temperatures would favor the reproduction of both ants and homopterans, but it seems unlikely that the ants would be capable of great colony expansion without the food derived from homopterans. Then in the first half of 2007, northern Florida experienced the worst drought since 1933 with many rainless weeks and severely depressed water tables. In August 2007, a walk-through survey of the experimental plots 228 (which had harbored such large populations in 2004–6) and 231 detected very few nests of *D. mariae*, although the ants were present on some plants that harbored homopterans.

These patterns, together with the strong association with homopterans suggest that the key life cycle feature of *D. mariae* may be the capacity for extremely rapid and opportunistic population increase that allows the ants to closely track populations of homopterans, whether the homopteran fluctuations are seasonal or stochastic. Many homopterans have the capacity for rapid increase of their populations, allowing rapid tracking of favorable conditions.

Like *D. mariae*, the Argentine ant *L. humile* also exhibits strong seasonal cycles with temporary nests near food sources, strong seasonal variation in queen number and winter contraction to one or a few central nests (Heller 2006a,b). This similarity suggests that such a life history is a syndrome, but just what habitat this syndrome is an adaptation for is not clear. *D. mariae* is a denizen of undisturbed longleaf pine habitat, whereas *L. humile* reaches its greatest densities in highly disturbed habitats, making it unlikely that the syndrome is a specific adaptation for disturbed habitats. One could argue that the capacity for rapid increase adapts a species to exploit any rich but ephemeral resource, be it homopteran blooms or disturbed habitat (which under natural circumstances, is usually short-lived). Indeed, a large fraction of the invasive ants that colonize disturbed habitat are polygyne with the concomitant capacity for rapid population increase, and many depend heavily on homopterans (Porter and Savignano 1990; Tsutsui and Suarez 2003).

Several other dolichoderine ants have strong dependence on homopteran-derived honeydew, including several species of *Dolichoderus* and several species of *Azteca*(Beattie 1985; Davidson and McKey 1993; Johnson 2001; Davidson et al. 2004), but none of these have been identified as being invasive. Seasonal polydomy is also not restricted to invasive or polygyne ants. Some species, no matter what their gyny, show seasonal polydomy, fractionating in the spring and coalescing again in the fall. Examples include the monogyne *Myrmica punctiventris* (Snyder and Herbers 1991; Banschbach et al. 1997) and *Leptothorax tuberointerruptus* (Partridge et al, 1997), and the polygyne *Linepithema humile* (Heller 2006a,b) and *D. mariae*. Even in polygyne species, some satellite nests may be queenless during the summer (Herbers 1984).

The progression from monodomous to polydomous social systems can lead to supercoloniality or unicoloniality. Supercoloniality consists of very large polydomous polygyne colonies that are hostile to other supercolonies. For instance, in its native range in South America, mature colonies of the polydomous, polygyne Argentine ant *L. humile* occupy very large territories and engage in territorial conflict at their boundaries (Holway 2004). The colonies of *D. mariae* that were observed seem too small to be called supercolonies. Unicoloniality, the complete absence of hostility on a geographic scale, describes a huge vastly dispersed population without hostility among occupants of different nests (Crozier and Pamilo 1996; Giraud et al. 2002). In the opinion of the junior author (the senior author is not invested in this debate), unicoloniality simply means that functional social organization does not involve hostility among units. It does not mean that the “unicolony” is a single, functional unit, an impossibility under any circumstance. The findings of Heller (2006a,b) with Argentine ants demonstrate that functional units are not necessarily recognizable by hostility among the units. A similar situation probably exists in the polygyne form of *S. invicta* (Goodisman 1996), where hostility is essentially absent, but nests are functional units, with little evidence for exchange of workers or queens among nests.

With respect to polydomy in general, most ant colonies are central place foragers, that is, foragers emanate from the nest and return to it with their booty (e.g. Bourke and Franks 1995). Clearly, even in the absence of competitive neighbors, distance and travel time, exposure to predation, heat and desiccation, and possibly orientation capacity, limit the exploitation of the space around the nest. At least in theory, ant colonies that are able to break up into separate units ought to be better able to exploit a larger area. Such a break-up into polydomy ought to be eased when there are multiple queens, as in *D. mariae* and *L. humile*, but it is clear that polygyny is not a necessary condition for polydomy.

Comparison of the daily production of eggs with the daily birth of adult workers is an estimate of egg survival. Egg survival was only 3 to 5% in July. The fate of 95 to 97% of the eggs merits further investigation. One possibility is that the queens lay trophic eggs, as do fertile *Myrmica* queens (Wardlaw and Elmes 1995), and that these are fed to the developing larvae or the queens. An alternate possibility is that there exists an intense competition among queens to have their eggs reared into adult ants, causing extreme egg attrition. This issue needs to be investigated.

### The effect of fire

These studies in both areas started immediately after a prescribed fire had burned away the groundcover. Since *D. mariae* has shallow nests, above-ground trails and a dependence on homopterans on low plants (e. g. bracken ferns and runner oak), it seems likely that a ground fire, especially during the active season, would be devastating to this ant. It is possible that the initially small size and large increase in the number of active nests in colonies 231-1 and 228-1 from year 2004 to year 2005 represents recovery from the prescribed burn. However, militating against this conclusion is the fact that other colonies remained the same size or even decreased in the second year after the fire. It is likely that the increase in number of active nests found at the base of food sources from year 2004 to 2005 represents recovery from the prescribed burn. Most of the low plants (e.g. bracken ferns and runner oak) that were devastated by the fire did not recover to more or less full biomass until the second survey year, but it is also possible that young plants sustain larger populations of homopterans. The nature and extent of the effects of fire on *D. mariae* colonies need more investigation.

### Colony reproduction

Although there are no data on the subject, it is interesting to speculate on how new colonies of *D. mariae* come into being. Perhaps if colonies contract to two widely-separated colonies during a winter, there is a fair chance that they will expand in different directions the following spring. If the expanding colonies remain unconnected long enough, they may acquire distinct colony odors that prevent them from fusing again, should they meet. Another possibility is that large *D. mariae* colonies become fragmented by fire, remaining separate long enough to become distinct colonies. Such questions could probably be addressed with molecular genetic methods.

Our estimates of sexual production in *D. mariae* were lower than those of Talbot's (1956) study of nuptial flight in Missouri. Perhaps the peak of sexual production in the colonies we studied occurred earlier or later than the July sample, or these colonies were less productive than Talbot's. Our samples were not adequate for estimating sexual production.

### Polygyny in D. mariae

Contrasted with monogyny, the consequences of polygyny are said to include higher population densities, lack of territoriality, low alate and high worker production, low seasonal and lifetime colony size variation, and small workers (Hölldobler and Wilson 1990). Whereas *D. mariae* has high population densities and high worker production rates, it displays high seasonal and lifetime colony size variation, fairly high sexual production and territoriality. The high seasonal and lifetime colony size variation of *D. mariae* share elements with those reported for *M. punctiventris* by Snyder and Herbers (1991) and the Argentine ant *L. humile*, in particular, ant colonies that experience seasonal polydomy. However, most ants that are polygynous do not display seasonal polydomy.

### Nest architecture

Compared to other ground-nesting ants, the nests of *D. mariae* can best be described as peculiar because they lack most of the elements common to the nest architecture of other species. These elements include more or less vertical tunnels connecting more or less horizontal chambers and top-heaviness (i.e. a larger proportion of the chamber area is near the ground surface, rather than at depth) (Tschinkel 1987; 2003; 2004; Mikheyev and Tschinkel 2004). Beneath wiregrass clumps, the ants build a single large conical chamber that is criss-crossed by the grass's fibrous roots to produce scaffolding that the ants use to arrange their brood, workers, queens, and alates. There thus seems no opportunity for the colony to organize spatially by segregating ants among different chambers, or to regulate working-group size by means of chamber size, as has been suggested by Tschinkel (2004) for ants that produce the more common types of nest architecture. Brian (1956; 1983) and Porter and Tschinkel (1985) showed that group size affected brood rearing efficiency in *Myrmica rubra* and *S. invicta*. How are the interests of efficiency served in *D. mariae* nests? The absence of clear morphological castes or meaningful size variation among the workers intensifies this question.

In addition to nesting behavior, Wheeler (1905) and Cole (1940) also described the presence of thatch, noting that the ants often covered the mounds with pine needles and leaves. Similar behavior has also been documented in *D. mariae's* congeners *D. plagiatus* (Cole, 1940), *D. pustulatus* (Wesson and Wesson, 1940), and *D. taschenbergi* (Trager, personal communication).

### Feeding biology

Within their territory, the ants follow and exploit their food source of honeydew provided by different Homopterans. The ants seem not to be very specific to particular homopterans or host plants. As the seasons change and different Homopterans arise on different host plants the ants exploit these in turn by forming nests at the base of host plants. Possibly polydomy is an adaptation to exploit the dispersed and rapidly changing populations of Homopterans, which in turn allows polygyny and extreme colony growth rates. Wilgenburg and Elgar (2007) found polydomous social insects might reduce the costs of foraging by the strategic distribution of nests throughout their territory or home range. They showed a positive correlation in the meat ant, *Iridomyrmex purpureus*, between the maximum distance between trees containing homoptera and the maximum distance between nests within a colony. They proposed that this pattern may arise if new nests are built nearer to trees containing homopteran populations.

The availability of homopterans may explain the anomalous difference in the number of active nests in colony 231-1. Whereas this colony had only one nest in January 2005, in January 2006 it had 18 active nests, 10 of which were located at the base of host plants (mostly saw palmetto) harboring pseudococcids. On the other hand, this anomalous difference could have been a result of exceptionally warm temperature (highs 23°C and lows 14°C) during the January 2006 survey. These exceptionally warm temperatures could have induced behaviors typical of the spring (oviposition and worker production), and might also have stimulated homopteran populations. Perhaps it is a combination of temperature and homopteran tending.

### Spatial aspects

*D. mariae* populations are extremely local. Large areas of Apalachicola National Forest were searched before *D. mariae* study populations were found. However, where populations do occur, the ant carpets the landscape. Colonies persist for many years; the population in compartment 228 was discovered by WRT in the mid-1970s. Because a large fraction of the colony was on the surface foraging, true colony size is probably much larger than was estimated, possibly up to several million ants. The area occupied by the colony is similar year to year even though the colony contracts down to one or two nests in the winter. This constancy suggests territoriality, and indeed, territorial battles were observed where workers from different colonies met. When workers from different colonies were mixed, they fought, which suggested that the colonies defend an absolute territory. Even with strong territoriality, no pattern in the position of over-wintering nests was found. Among the survey colonies, some over-wintering nests were either closest to, or farthest from colony territory boundaries.

### Ecological impact of D. mariae

*D. mariae* may have considerable ecological importance to the longleaf pine ecosystem. First, since colony size may exceed several million workers, they probably play a substantial role in energy flow, with enormous numbers of ants sucking honeydew from enormous numbers of homopterans, that draw energy from their food plants. Homopteran populations are usually sparse and have little impact on the net growth of plants, but if conditions permit, populations can become extremely large, in which case they do affect plant growth (Townsend 2000). *D. mariae* possibly regulates homopteran cluster sizes by moving homopterans to additional host plants (Aphidiidae, Laskis, personal observation). The ants protect the homopterans from “sooty-mold”, a fungus that grows on the honeydew and causes the surface of the leaves to turn black, reducing photosynthesis (Townsend 2000; Laskis, personal observations).

Since the abandoned nests retain their conical shape, they provide shelter for a variety of animals. During the spring field season, snakes begin to emerge after winter hibernation, and several snakeskins were removed from several abandoned nests. These abandoned nests also provided shelter for ground dwelling spiders and small vertebrates such as lizards. Currently the only other animal of the long-leaf pine forest known to provide soil shelter for other animals is the gopher tortoise (Myers and Ewel 1990; Whitney and Means 2004).

### Caveats

The methods of this study were relatively crude. As a result, the quantitative description of the life cycle should be considered a rough approximation. Moreover, population estimates did not include foragers outside the nest. We applied a single year's census data to both years, creating the implied assumption that the nest census patterns would have been similar in the second year. Obviously, this is not necessarily so. Variation of census values among nests was high, probably obscuring some patterns, but all reported patterns were significant at least at the 5% level. Because we applied census values from a single set of nests each season to both colonies, the patterns at the colony level were driven mostly by the number of nests (an observation of high reliability). Surveying colonies only 4 times a year no doubt missed some interesting patterns, including details of the schedule of sexual production, queen adoption and worker/queen die-off, but labor availability foreclosed more samples. The interesting patterns we uncovered in spite of these limitations suggest that *D. mariae* holds many secrets worth uncovering.
